# Sensory-Motor Mechanisms Increasing Falls Risk in Diabetic Peripheral Neuropathy

**DOI:** 10.3390/medicina57050457

**Published:** 2021-05-08

**Authors:** Neil D. Reeves, Giorgio Orlando, Steven J. Brown

**Affiliations:** Research Centre for Musculoskeletal Science & Sports Medicine, Departments of Life Sciences and of Sport and Exercise Sciences, Faculty of Science and Engineering, Manchester Metropolitan University, Manchester M1 5GD, UK; G.Orlando@mmu.ac.uk (G.O.); S.Brown@mmu.ac.uk (S.J.B.)

**Keywords:** diabetic neuropathy, balance, unsteadiness, walking, diabetic foot ulcer, falling, foot, ankle

## Abstract

Diabetic peripheral neuropathy (DPN) is associated with peripheral sensory and motor nerve damage that affects up to half of diabetes patients and is an independent risk factor for falls. Clinical implications of DPN-related falls include injury, psychological distress and physical activity curtailment. This review describes how the sensory and motor deficits associated with DPN underpin biomechanical alterations to the pattern of walking (gait), which contribute to balance impairments underpinning falls. Changes to gait with diabetes occur even before the onset of measurable DPN, but changes become much more marked with DPN. Gait impairments with diabetes and DPN include alterations to walking speed, step length, step width and joint ranges of motion. These alterations also impact the rotational forces around joints known as joint moments, which are reduced as part of a natural strategy to lower the muscular demands of gait to compensate for lower strength capacities due to diabetes and DPN. Muscle weakness and atrophy are most striking in patients with DPN, but also present in non-neuropathic diabetes patients, affecting not only distal muscles of the foot and ankle, but also proximal thigh muscles. Insensate feet with DPN cause a delayed neuromuscular response immediately following foot–ground contact during gait and this is a major factor contributing to increased falls risk. Pronounced balance impairments measured in the gait laboratory are only seen in DPN patients and not non-neuropathic diabetes patients. Self-perception of unsteadiness matches gait laboratory measures and can distinguish between patients with and without DPN. Diabetic foot ulcers and their associated risk factors including insensate feet with DPN and offloading devices further increase falls risk. Falls prevention strategies based on sensory and motor mechanisms should target those most at risk of falls with DPN, with further research needed to optimise interventions.

## 1. Introduction

Diabetes and its long-term complications are considered a global public health emergency linked to high levels of morbidity, disability and mortality. Recently, the International Diabetes Federation estimated that half a billion people suffer from diabetes, which corresponds to 5.3% of the global population [1]. Diabetic peripheral neuropathy (DPN) is the most common and debilitating complication of diabetes, affecting up to 50% of diabetic patients depending on their age and duration of diabetes [2]. This condition is characterised by marked axonal degeneration and segmental demyelination, which affects the peripheral sensory (small and large nerve fibres) and motor nerves [3]. Consequently, patients may present with impaired peripheral sensation and marked deterioration of muscle morphology and function.

Diabetes is associated with multiple complications and perhaps because of the number of potential complications, falls in patients with diabetes is a clinical issue that is only now beginning to gain broader recognition. Falls in the general ageing population is an internationally recognised clinical problem, with one in three people over 65 years falling each year [4]. Falls in people with diabetes is actually an even greater clinical challenge—someone with DPN is up to 20-fold more likely to fall compared to an age-matched non-diabetic control [5,6]. Studies have established DPN as a strong independent predictor of falls risk [7,8,9,10,11]. The clinical implications of DPN-related falls stretch well beyond the injurious consequences to include psychological distress [12], fear of falling [13] and curtailment of physical activities linked to unsteadiness [14]. 

DPN-related unsteadiness is common, with a large-scale UK-USA study showing that 23% of the 484 patients with DPN perceived themselves as being unsteady, with associated balance problems [15]. One of the symptoms most strongly predicting depression in people with diabetes is DPN-related unsteadiness [12,15,16,17], highlighting its key role in psychological distress. Patients do not always self-report their unsteadiness as a clinical problem because they do not identify it to be caused by DPN, but rather incorrectly attribute it to diminishing capacities as a result of ageing [12]. In fact, many DPN patients affected by unsteadiness and falls are much younger than where ageing- and frailty-related falls become prominent in the general ‘ageing’ population. This patient misconception and lack of reporting leads to falls often being neglected as a clinical issue by both patients and clinicians. 

**Figure 1:**
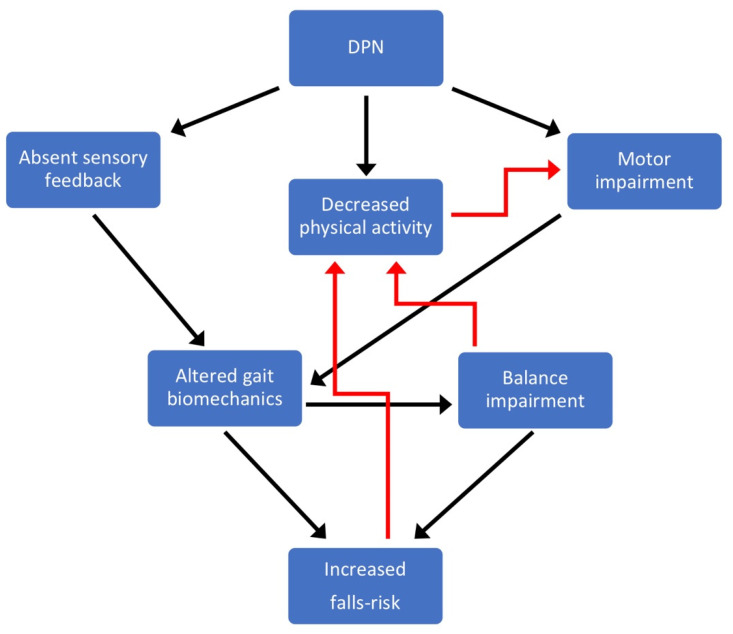
Schematic diagram linking the underlying sensory-motor causes of increased falls risk with diabetic peripheral neuropathy (DPN). Briefly, DPN causes lower limb motor impairments and a loss of sensory feedback in the feet that alter gait biomechanics, leading to balance impairments, ultimately resulting in an increased falls risk. Balance impairments and the increased falls risk due to DPN increase the perception of unsteadiness and fear of falling, leading to a reduction in physical activity. Reduced muscular loading due to a curtailment of physical activity will also compound DPN-related muscular weakness with further motor impairments, causing further alterations to gait and subsequent balance impairment and increased falls risk.

This review explores the sensory and motor mechanisms associated with DPN underpinning altered gait biomechanics, which cause balance impairments and increase falls risk in patients with DPN. The schematic diagram presented in Figure 1 provides a framework for this review, supported by evidence described in the sections below. Briefly, this diagram links how DPN causes lower limb motor impairments and a loss of sensory feedback in the feet. These two factors (motor impairment and sensory loss) cause altered gait biomechanics, leading to balance impairments, ultimately resulting in an increased falls risk. Balance impairments and the increased falls risk due to DPN increase the perception of unsteadiness and fear of falling, leading to a reduction in physical activity. Reduced muscular loading due to a curtailment of physical activity will also compound DPN-related muscular weakness with further motor impairments, causing further alterations to gait and subsequent balance impairment and increased falls risk.

## 2. Sensory-Motor Pathophysiology and Risk Factors for Gait Impairment in Diabetes

Peripheral sensory loss in the feet and motor system deterioration are consequences of DPN and important risk factors for altered gait biomechanics, balance impairment and increased falls risk. Although other risk factors including cognitive function, centrally-acting medications and psychological factors will also undoubtedly play a role in increasing falls risk in the diabetes population, this review primarily focusses on sensory and motor deficits as key falls risk factors, reflecting the proportion of available evidence in this area.

Sensory loss in the feet causes alterations to gait biomechanics, affecting balance, and this is a major falls risk factor [18,19]. Accurate assessment of DPN is therefore important for understanding and stratifying falls risk in research. Currently, numerous tests and scoring systems have been proposed for the diagnosis and staging/stratifying of DPN [2]. As sensory deficits are most prominent in DPN, most testing/scoring systems are focused on exploring the different aspects of peripheral sensory function, such as pain sensitivity, proprioception, pressure and vibratory perceptions [20]. The Toronto consensus statement recommends as criteria for the diagnosis of DPN the combination of abnormalities in nerve conduction studies and the presence of one or more signs or symptoms [21]. The presence of only symptoms and/or signs defines a *probability* of having DPN, rather than a certainty. The modified neuropathy disability score (NDS) tests a range of different sensory modalities at the foot and ankle—(i) vibration perception (with a 128 Hz tuning fork), (ii) temperature perception (warm/ cold), (iii) pain (sharp/ blunt) and (iv) the Achilles tendon reflex [22]—providing a score between 0 and 10, with 0 demonstrating intact sensation and 10 indicating a complete absence of sensation due to DPN. The vibration perception threshold (VPT) is a semi-quantitative measure of sensory perception, typically applied at the apex of the Hallux, measured using a neuro- or biothesiometer. The VPT provides a value between 0 and 50 Volts, with 50 Volts indicating a complete absence of sensation with DPN. Severe DPN is typically stratified by a modified NDS score of ≥6, or a VPT of ≥25 Volts [23]. Other techniques to assess the presence of DPN include electromyography and corneal confocal microscopy [24,25] that require high costs, a long duration and a highly qualified operator. 

Diabetes is responsible for a deterioration of muscle strength, which becomes more severe with the development of DPN [26] and is responsible for altered gait biomechanics, impaired balance and increased falls risk (Figure 1). Muscle strength is impaired at the initial stage of the disease and progresses with chronic hyperglycaemia [27,28,29]. Several muscle groups of the upper and lower body are involved, although the major detrimental effects are documented at the proximal (knee flexors and extensors) and distal (ankle dorsal and plantar flexors) segments of the lower limb [26]. These deficits are particularly evident during high angular velocities (isokinetic muscle testing), indicating an impairment of muscle power (i.e., force x velocity) [30]. Patients with DPN exhibit a more severe strength impairment of foot (intrinsic foot), ankle and knee muscles because of the motor nerve damage [31]. Studies have reported ~8% to 15% lower strength levels in non-neuropathic diabetic patients compared with age-matched healthy individuals at both knee and ankle muscles and deficits ranging between 25% and 45% in those diabetic patients developing DPN [24,30,32,33]. There is also evidence showing that muscle strength decreases in relation to the severity of motor nerve damage [31].

Muscle morphological abnormalities are a further typical feature of diabetes and DPN. These alterations include loss of muscle mass and an accumulation of intra and intermuscular fat [24,32,34,35]. Longitudinal studies indicate that diabetes patients manifest an accelerated loss of muscle mass, which is independent from long-term diabetic complications [36,37,38]. In particular, diabetic patients are shown to experience a 2-fold greater decline in quadriceps muscle mass compared with non-diabetic individuals [37]. In patients with DPN, loss of contractile tissue and fat infiltration are hallmarks of motor nerve dysfunction [31]. These deficits occur early in the feet and progress in the lower legs, but only as late-stage complications [39,40]. Furthermore, evidence shows a marked decline in annual muscle volume of the intrinsic foot muscles (DPN, 3% vs. non-neuropathic diabetes, 1%) and ankle dorsal and plantar flexors (DPN, 4% to 5% vs. non-neuropathic diabetes, 2%) in patients with DPN compared with non-neuropathic subjects [40]. In one of our studies, we documented a 20%–22% lower quadriceps muscle volume and 10% greater intramuscular fat in the ankle plantar flexor muscles in a mixed group of diabetes patients with and without DPN compared to non-diabetic controls [41]. Our results indicate that patients with DPN are characterised by muscle morphological abnormalities which affect not only the most distal parts of the lower limbs, but also more proximal lower limb muscles. [24]. 

Although the decrease in muscle strength may be ascribed to lower muscle mass, it is important to note that diabetes and DPN patients are characterised by poor muscle quality (i.e., strength/force produced per unit of muscle area) [28,35]. Non-enzymatic glycosylation (glycation) of skeletal muscle proteins, sarcoplasmic reticulum dysfunction and mitochondria abnormalities have been proposed as the main factors underlying this deficit [26,42]. In particular, glycation appears to induce structural modifications of the motor proteins (actin and myosin) responsible for impairments in the force- and power-generating capacity of contractile muscle fibres [43,44]. Patients with DPN are also characterised by progressive axonal degeneration and segmental demyelination of the peripheral motor nerve [3]. These deficits cause a marked loss of motor units and neuromuscular transmission impairment [31,45] Notably, it has been estimated that patients with severe DPN may display up to a 60% lower number of motor units in the leg muscles compared with non-diabetic individuals [46]. Altogether, these deficits lead to a severe deterioration in muscle mass and quality, and increases to intramuscular non-contractile tissue. 

## 3. Gait Characteristics in Diabetes and DPN

Alterations to the natural walking strategy including a slower walking speed, reduced joint range of movement and lower joint moments have been shown to occur even before the onset of measurable DPN [47,48,49,50]. Slower self-selected speed of walking (i.e., the speed at which people naturally choose to walk) is one of the most established findings, with non-neuropathic diabetes patients walking slower compared to aged-matched matched controls without diabetes, and those with DPN walking even slower [25,48,51,52,53,54,55,56,57,58,59,60]. Natural walking speed is an important measure due to its ability to be impacted by any and all of the gait mechanisms within an individual that will be discussed below.

**Figure 2:**
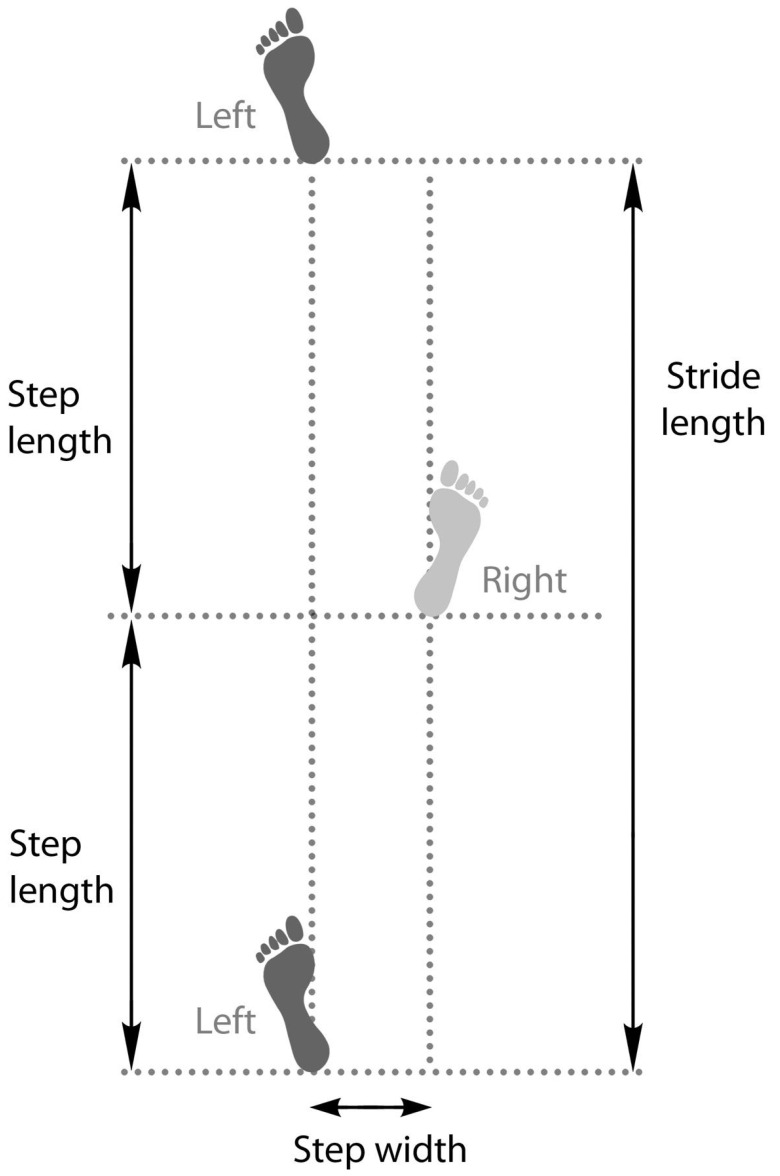
Diagram to illustrate the gait cycle parameters of stride length, step length and step width.

**Figure 3:**
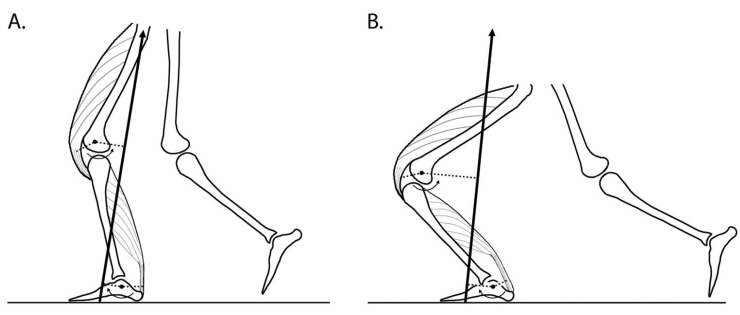
Schematic diagram to illustrate how strep length and increased joint flexion influence the joint moments (rotational forces developed around joints). The black vertical arrow indicates the ground reaction force and the dotted lines indicate the external and internal moment arms; defined as the perpendicular distance between the joint centre [black dot] and the ground reaction force [external moment arm] and between the joint centre and the action line of the tendon [internal moment arm]. In (**A**) with a shorter step length the external moment arms around the knee and ankle are shorter compared to the situation in (**B**) with a longer step causing greater knee and ankle flexion, thereby increasing the external moment arm length and the magnitude of the joint moments around these joints (as a proxy for the muscular forces).

Step length is the anterior–posterior distance between left and right consecutive foot placements, with stride length being the distance between the floor contact points of the same foot within a single gait cycle (Figure 2). Patients with diabetes walk with a shorter step and stride compared to non-diabetic aged-matched controls [48,51,53,54,56,58,59,61,62]. The reason for this likely relates to a reduction in the muscular demands of walking. Shorter strides require less flexion of the lower limb joints. For example, the knee joint undergoes less flexion during walking upon weight acceptance in patients with diabetes compared to non-diabetic controls [54,63,64]. Minimising flexion of the lower limb joints reduces the leverage (also known as the external moment arm) of the ground reaction force around that specific joint, which reduces the joint moment, thereby lowering the muscular forces required to create and control movement at the joint (Figure 3). 

Step width is the medio-lateral distance between feet during walking (Figure 2). Patients with diabetes and DPN typically adopt a wider step as they walk [52,59]. It has been suggested that a wider step during gait is used by diabetes patients to promote greater stability, as a larger stance provides a wider base of support during standing. However, walking involves a constant transition between the two feet contacting the ground and balancing on a single limb while progressing the other limb ahead. The body’s centre of mass therefore needs to sway from side to side towards each foot as it is placed onto the ground during walking. A wider step could therefore induce greater side-to-side sway, thereby compounding existing balance impairments and increasing unsteadiness in patients with diabetes and DPN. In line with this theory, a recent study showed a strong positive correlation between balance impairment and step width in a cohort with non-neuropathic diabetes and DPN [19]. Specifically, as step width increased, balance impairments increased, but it is unknown whether a wider step is a cause of this balance impairment, or one of the characteristics inherent to a population with balance impairments. 

### 3.1. Joint Motion and Joint Moments during Gait

Diabetes and DPN alters the range of motion across all lower limb joints during walking compared to controls [48]. Patients with non-neuropathic diabetes and DPN are consistently reported to move through smaller joint ranges of motion at the knee [54,63,64] and ankle [63,64,65] joints compared to controls. Less consistent range of motion findings are report for the hip, with some studies reporting reduced hip range of motion during gait in diabetes and DPN patients [48,64], whilst in other studies the hip is sometimes an exception, reported to move through a greater range of motion in diabetes patients [56]. A greater hip joint range of motion during gait in diabetes patients despite smaller knee and ankle joint ranges of motion is likely explained by compensatory mechanisms linked to the joint moments produced around these joints and the execution of a ‘hip strategy’ (see below). 

Diabetes patients have reduced lower-limb muscular strength (between 8% and 45% weaker) compared to age-matched non-diabetic controls [24,30,32,33,41], which is likely one of the key factors causing them to decrease their walking speed to reduce the joint moments and therefore reduce the muscular demands of walking. Patients with diabetes and DPN have been consistently reported to generate lower joint moments at the knee [25,49,50,56,60] and ankle [25,49,50,56,60,64] during gait compared to non-diabetic controls. At the hip, although lower joint moments in diabetes patients have been reported [56], the majority of gait studies report higher hip joint moments in diabetes patients [49,50,60,64]. Higher hip joint moments, with lower knee and ankle joint moments in diabetes patients is consistent with the ‘hip strategy’. Non-neuropathic diabetes patients and those with DPN tend to have a much less forceful ‘push off’ with the ankle plantar flexor muscles, evidenced by a lower ankle joint moment in this terminal stance phase of the gait cycle and a reduced ankle plantar flexion joint range of motion. This ‘push off’ movement emanating from the ankle plantar flexors is important for both maintaining the body’s momentum, and to propel the leg through the gait cycle so it can be extended out in front of the body to step forwards within each stride. To compensate for this reduced ankle plantar flexion propulsion, patients tend to use a ‘hip strategy’ first described in detail in this patient population by Mueller et al. [56], whereby they ‘pull’ the leg up from the floor with the hip flexor muscles and then extend the leg out in front to allow a forwards step, resulting in the greater hip joint moment described above, and sometimes a greater hip joint range of motion [56]. Visually, this strategy would be evident by the patient’s foot leaving the ground in a more neutral (‘flatter’) position as the leg is pulled up by the hip flexors. In contrast, the leg of a non-diabetic control would leave the ground via the ends of the toes as their ankle plantar flexor muscles generate higher forces (measured as a higher ankle joint moment) and transition the ankle into a much more plantar flexed position as the leg is ‘pushed’ or ‘propelled’ into the swing phase by the ankle plantar flexors. The ‘pull’ from the hip approach seen in diabetes patients also contributes to the smaller step and stride lengths described above, as it is a less efficient mechanism of propelling the leg through swing, but reduces the ankle joint moment demand and therefore the muscular force required from the plantar flexor group. 

Although lower joint moments in diabetes patients could be explained by a slower walking speed, a recent study highlighted that non-neuropathic diabetes patients and particularly those with DPN were able to reduce ankle joint moments compared to controls even while walking at the same speed [64]. In this cross-sectional study, participants walked at the same matched speeds across a range from slow (0.6 m/s) to fast (1.6 m/s) and despite this speed restriction, non-neuropathic patients and particularly those with DPN developed lower ankle joint moments compared to a group of non-diabetic controls. Despite no differences in foot length between the groups studied (DPN, non-neuropathic diabetes and controls), patients with DPN altered how the ground reaction force was applied on their foot, reducing the external leverage around the ankle joint centre and minimising forces developed by the ankle plantar flexor muscles [64]. This can be regarded as a natural gait strategy (regardless of whether this was conscious) to minimise these muscular forces while walking, compensating for marked muscular weakness in this cohort. At the knee joint, shorter steps taken during gait by patients with diabetes, combined with a ‘stiffer’ gait, reduce the flexion of the knee joint, in turn reducing the external moment arm of the ground reaction force around the knee joint centre, minimising the knee joint moment and the required forces from the knee extensor muscles (Figure 3). 

‘Operating strength’ can be quantified by relating the joint moments generated during gait to an individual person’s maximum strength capability for that same muscle group (tested under the same conditions between the gait task and maximum muscle testing). This provides an indication of how close people are operating relative to their maximum muscular capabilities during gait (see [66,67] for principles). Despite the generation of lower ankle and knee joint moments during overground and stair walking, non-neuropathic diabetes patients and patients with DPN have been shown to have higher ‘operating strength’, meaning their muscles are operating closer to their maximum capacity [49,50]. This brings with it potential risks, as people need to have some level of ‘strength reserve capacity’ to call upon for any unexpected situations such as a trip or perturbation to balance. The higher the ‘operating strength’ (and therefore the lower the strength reserve capacity), the higher the risk that any unexpected gait event, or perturbation to balance will overcome the muscular strength capabilities of the person and leave them unable to sustain balance, resulting in a fall (Figure 1). 

As indicated by the gait alterations described above, studies that have included three participant groups—(i) patients with severe DPN, (ii) non-neuropathic diabetes patients and (iii) aged-matched controls without diabetes—consistently show a graded response in terms of the extent of alteration to gait parameters, with gait parameters most severely affected in patients with DPN, followed by non-neuropathic diabetes patients and matched controls as the reference group. Gait alterations in non-neuropathic diabetes patients have also been reported in other gait-related variables including impaired visual gaze behaviour during gait [68], reduced stepping accuracy [68], reduced speed of strength generation [18], a higher metabolic energy cost of walking [69] and altered Achilles tendon function during walking [63]. 

### 3.2. Variability of Gait

Gait variability, the variation in movements from step to step, is a common assessment of gait ‘quality’, since greater variability can be indicative of diminished muscular and neurological control and associated with increased falls risk in the diabetes population [70]. Variability in both the temporal (timing of gait cycle events) and spatial (step length and width) gait parameters is higher for patients with DPN compared to controls [9,51,55,61,70] and is also indicative of greater instability [55]. Studies have also shown that this variability becomes much more pronounced when walking in challenging environments such as on uneven/irregular surfaces [9,61,70]. 

## 4. Diabetic Peripheral Neuropathy and Falls Risk

DPN has been shown as a strong independent predictor of falls risk [7,8,9,10,11], which is in line with findings from gait laboratory-based studies described above showing altered gait biomechanics and marked balance impairments in patients with DPN and reflecting both sensory and motor impairments (Figure 1). 

Self-perceived unsteadiness measured using two specific items on the neuropathy- and foot ulcer-specific quality of life instrument (NeuroQoL), has been shown to discriminate between patients with DPN from non-diabetic controls and notably, from non-neuropathic diabetes patients [71]. This is consistent with research showing that only patients with DPN show marked unsteadiness [19]. Diabetes patients’ perception of their unsteadiness actually matches gait laboratory-based measurements of balance impairments shown by strong correlations between self-perceived unsteadiness and gait laboratory measures of dynamic balance [71]. Substantial gait alterations shown in patients with DPN with greater self-perceived unsteadiness may suggest that they are not only aware of their unsteadiness but attempt to self-regulate this increased falls risk by slowing their gait and taking shorter steps [71]. This also has important clinical implications, because simply asking patients with DPN how unsteady they feel using a sensitive scale (NeuroQoL, [72]) provides a quick and relatively accurate indication of balance impairments and falls risk. 

One of the major reasons why DPN has been shown to be an independent risk factor for falls is due the complete absence of peripheral foot sensation and the delayed neuromuscular control this causes during gait (Figure 1). Insensate feet due to DPN cause a slower speed of strength generation [18], reducing lower limb control over gait, leading to impaired balance [19]. The speed of strength generation requirement from the lower limb muscles is higher on stairs compared to overground walking and balance impairments are greater, indicating the heightened sensory and muscular demands and therefore elevated falls risk for DPN patients on stairs compared to overground walking [18,19,71]. Patients with DPN identified as fallers have shown a greater step time variability on a challenging surface compared to those with diabetic neuropathy who had not fallen over a one-year follow up [9,70]. 

### 4.1. Peripheral Sensory Loss and Falls Risk

Although often difficult to untangle the effects of sensory and motor neuropathy on gait biomechanics and falls risk, the absence of peripheral foot sensation (insensate feet) due to DPN plays a crucial role (Figure 1). Sensory feedback from the feet during walking is fundamental to activating the relevant muscles to stabilise the lower limbs and control balance, and when absent in patients with DPN this has been shown to alter gait biomechanics [18] and impair balance [19] increasing falls risk. This is also supported by gait changes seen in studies of healthy individuals with experimentally induced plantar insensitivity, exhibiting characteristics of DPN gait including slower walking speed and altered foot loading [73,74]. In DPN patients, a slower ‘speed of strength generation’ in the immediate period following foot–ground contact has been reported during overground and stair walking and largely attributed to the absence of sensory feedback resulting from insensate feet [18]. This same study also showed how the timing of lower limb muscle activations was altered in patients with DPN, again attributed to the absence of sensory feedback from the feet. The result of these changes in gait biomechanics arising from absent foot sensory feedback is reduced stabilisation by the lower limbs, leading to balance impairments [19,71](Figure 1). Balance has been quantified by measuring the medio-lateral (side-to-side) separation between two points—(i) the body centre of mass and (ii) the centre of pressure under the feet—and has been termed as ‘dynamic sway’ in the medio-lateral direction. Although the body centre of mass and the centre of pressure under the feet are both constantly moving points that fluctuate during walking, their separation needs to be regulated within certain limits and if they separate too far towards, or exceed these limits, then people will need to take evasive action, or will sustain a fall. Patients with DPN were shown to have marked balance impairments as measured by increased dynamic sway [19]. A key finding from this study was that no balance impairments were observed in non-neuropathic diabetic patients (compared to aged-matched non-diabetic controls), highlighting the key role of sensory impairment arising from DPN in balance impairments and heightened falls risk (Figure 1). Furthermore, self-perceived unsteadiness remains unaffected by diabetes before the onset of DPN, but is significantly elevated in patients with DPN [71]. These self-reports are also consistent with gait laboratory measurements of balance, where only patients with DPN have been reported to show marked impairments to balance during walking, whereas balance was similar between non-neuropathic diabetes patients and non-diabetic controls [19].

### 4.2. Diabetic Foot Ulcers and Falls Risk

Diabetic foot ulcers are a major complication of diabetes, with up to one in three patients with diabetes developing a foot ulcer in their lifetime [75]. Infection and amputation are serious consequences associated with diabetic foot ulceration, with as many as ~33% progressing on to require amputation [75,76,77]. Multiple risk factors contribute to the development of diabetic foot ulcers, with one of the most significant being insensate feet due to DPN, ultimately culminating in sustained high pressure on the plantar foot surface (for review see Chatwin et al. [78]). In addition to the direct consequences, patients with a diabetic foot ulcer have been shown to be at a greater risk of falls. In a retrospective study of ~44,000 diabetes patients, those with a diabetic foot ulcer were twice as likely to fall (Odds ratio = 2.26) compared to patients who had not suffered a diabetic foot ulcer [79]. In line with this, the risk of fracture was 3-fold higher in those with a diabetic foot ulcer. Potential reasons for this increased falls risk, include the presence of associated risk factors for diabetic foot ulceration including, most notably, insensate feet due to DPN. Indeed, in this study, 23% of those with a diabetic foot ulcer had DPN, compared to only 6% in those without a foot ulcer [79], highlighting the central role of DPN and sensory loss to falls risk (Figure 1). In a study of 400 patients with a previous history of diabetic foot ulcers, 63% of patients experienced a fall over a two-year follow-up period [80]. The fallers had more comorbidities (82% of fallers vs. 66% of non-fallers) and in particular a greater proportion had insensate feet (62% of fallers vs. 52% of non-fallers) compared to those who did not fall over this two-year period. As a means of comparison, after one-year follow up, 54% of patients with a previous diabetic foot ulcer history had fallen [80], which is much higher compared to ~33% of the general population over the age of 65 years who fall annually [4], despite the population with a history of diabetic foot ulcers being younger (mean age 62 years). 

An active diabetic foot ulcer necessitates patients wearing an offloading device, which could be an important risk factor for falls when patients are ambulatory due to increased weight and potential limb length discrepancies. Postural instability has been identified as a key barrier in adherence to offloading devices in patients with a diabetic foot ulcer [14]. Offloading devices are purposely designed to limit the range of movement within the joints of the foot and around the ankle joint, facilitating offloading of the affected ulcer area. Although ulcer offloading is the treatment goal, patients will need to remain ambulatory to perform tasks of everyday living, but patients’ reluctance to wear these devices because of feelings of unsteadiness [14] suggest an elevated falls risk. Wearing an offloading device does markedly affect gait and stability of patients with an active diabetic foot ulcer, but reducing the height of devices (knee, ankle and shoe levels) can minimise these gat disturbances [81]. 

### 4.3. Sensory-Motor Falls Interventions 

Falls prevention strategies have primarily focussed on the motor system component through increasing the strength of lower limb muscles [68,82,83]. A recent study has targeted both motor and sensory components through the combination of exercise and visual gaze training [84]. This is based on the concept that visual gaze behaviour directs movement and therefore improving eye movements can help to improve gait and reduce the risk of falls [68,84]. Given the important role of insensate feet due to DPN in falls risk, future falls prevention strategies might seek to explore interventions that can help enhance detection of foot–ground contact during walking.

## 5. Conclusions

Sensory and motor deficits caused by DPN underpin changes to gait that cause balance impairment and increase falls risk. Muscle weakness and atrophy are prevalent in diabetes patients, but most marked in those with DPN compared to their non-neuropathic counterparts and evident in both distal and proximal regions of the lower limb. Insensate feet with DPN play a fundamental role in gait alterations, leading to increased falls risk, and largely explain the high falls risk in patients with a history of diabetic foot ulcers. Although changes to gait are consistently reported in diabetes patients prior to the onset of DPN, such alterations are far more marked in patients with DPN due to insensate feet and the extent of motor impairments. Importantly, gait alterations only reach a level where they markedly impair balance in patients with DPN, explaining why DPN is an independent predictor of falls risk and why patients with DPN are up to 20-fold more likely to fall compared to an age-matched non-diabetic control. The clinical implications of DPN-related falls stretch well beyond the injurious consequences to include psychological distress, fear of falling and curtailment of physical activities linked to unsteadiness. Positive results from falls prevention studies based upon targeting both sensory and motor risk factors have been reported. However, further research is needed to optimise interventions, particularly for strategies to compensate for the DPN-related sensory deficits. 

## Data Availability

Not applicable.
